# Management of Otogenic Meningitis: A Proposal for Practical Guidelines from a Multicenter Experience with a Systematic Review

**DOI:** 10.3390/jcm13185509

**Published:** 2024-09-18

**Authors:** Alessia Rubini, Guglielmo Ronzani, Edoardo D’Alessandro, Daniele Marchioni

**Affiliations:** 1 Department of Otorhinolaryngology and Head and Neck Surgery, University Hospital of Modena, 41125 Modena, Italy; rubini.alessia@aou.mo.it (A.R.); dalessandroedoardo@gmail.com (E.D.); daniele.marchioni@unimore.it (D.M.); 2 Department of Otorhinolaryngology and Head and Neck Surgery, University Hospital of Verona, 37134 Verona, Italy

**Keywords:** acute otitis media, meningitis, intracranial complications of otitis media, mastoidectomy, temporal bone, infective middle ear disease

## Abstract

**Background:** Otogenic meningitis represents the most common and life-threatening complication of infective middle ear diseases. However, no guidelines are available to describe the optimal management strategy and the role of surgical intervention. **Methods:** A six-year multicenter retrospective study on consecutive patients treated for otogenic meningitis caused by acute otitis and re-exacerbation of chronic otitis at the University Hospital of Verona and Modena was performed, and a systematic review regarding acute otitis media-related meningitis in accordance with the PRISMA 2020 statement was then conducted. **Results:** From the clinical chart analysis, 16 patients with surgical indications according to our decision-making flow chart were reviewed, with most of them undergoing surgery within 7 days of admission (*n* = 13, 81%). The systematic review ultimately utilized 24 studies (16 case reports and 8 case series) published between 1990 and 2023, with the overall analysis involving a total of 181 patients. **Conclusion:** The primary treatment for acute bacterial meningitis relies on antibiotic therapy, with surgical intervention being employed in the event of complications and when the initial treatment is not effective within 48 h. The objective of surgery is to sterilize the tympanic and mastoid cavity, thereby eradicating the suspected infective foci and managing any eventual intracranial complications.

## 1. Introduction

Intracranial otogenic complications, though rare, are fearsome consequences of some middle and inner ear diseases. Otogenic meningitis is the most reported example, representing 35–46.4% of all intracranial complications by itself [1,2]. According to some authors, its incidence is about 0.42 per 100,000 per year in the UK [2]. Pathogens disseminate from ear structures to the meninges through various routes: bloodstream transmission, anatomical connections linking the inner ear structures to the meninges, pathological conduits between the middle ear and the middle cranial fossa, and, in cases of thrombophlebitis, the blood vessels. Most frequently, its causes include acute and chronic otitis with or without cholesteatoma, traumas, post-operative onset (e.g., post-cochlear implantation, post-schwannoma removal, etc.), and inner ear malformations (e.g., incomplete partition type I, oval window perilymph fistulas, congenital temporal meningoceles, stapes footplate malformations, etc.) [3,4,5,6,7,8]. Some comorbidities, such as diabetes and acquired or congenital immunodeficiency, increase the risk of otogenic meningitis.

As infections traverse from the ear towards the central nervous system, pathogens from the notorious trio (i.e., S. pneumoniae, M. catarrhalis, and H. influenzae) act as the predominant etiological agents in otogenic meningitis. Despite the introduction of pneumococcal conjugate vaccines, S. pneumoniae still remains the most commonly involved [9].

On the other side, polymicrobial anaerobic meningitis is very rare nowadays, though it is related to a higher rate of sequelae and mortality, accounting for only 2.4% of all bacterial meningitis [10,11,12].

Clinical examination might not be helpful, as otogenic symptoms like otalgia and otorrhea may or may not be found, and in some instances, the otogenic source is only identified upon conducting a radiological assessment. Cerebrospinal fluid culture is the gold standard for diagnosis, and it should be performed as soon as possible, promptly followed by the initiation of empirical antibiotic therapy [9].

In this scenario, the role of surgery remains a topic of debate. While drainage of the infection source appears beneficial for expedited recovery and preventing bacterial dissemination, in the majority of cases, medical therapy alone is sufficient to achieve a full recovery. Moreover, even when it is performed, the proper timing of eventual surgery also remains unclear, as some authors suggest prompt surgery, while others recommend 48 h watchful waiting while on antimicrobial therapy before considering any surgical treatment [13].

To date, no guidelines concerning the role of surgery in the management of otogenic meningitis are available in the literature. Hence, the present paper aims to understand whether surgery is always needed in combination with antimicrobial therapy or, if it is not mandatory, what the proper indications for it are and how long it can be delayed following the onset of medical therapy.

In this article, we describe our experience through a multicenter case series on the surgical management of otogenic meningitis caused by acute ear infections and the re-exacerbation of chronic ones. Furthermore, we performed a systematic review of the literature focusing on the role of surgery within the management of acute otitis media-related meningitis.

## 2. Materials and Methods

### 2.1. Our Surgical Experience in Otogenic Meningitis

A multicenter retrospective chart review of consecutive patients treated for otogenic meningitis from January 2017 to December 2023 at the University Hospital of Verona and Modena was performed. Meningitis developing as a result of acute otitis media or an acute flare of chronic otitis media was taken into account, and data related to diagnostic management as well as the treatment modalities were collected. The present series included both adult and pediatric patients presenting with otogenic meningitis as their initial diagnosis, with indication of surgical treatment. Meanwhile, subjects with otogenic meningitis from causes other than acute otitis or the re-exacerbation of chronic otitis (i.e., post-surgical, due to inner ear malformations, etc.) and treated with medical therapy were excluded.

### 2.2. Our Decision-Making Process

**Figure 1:**
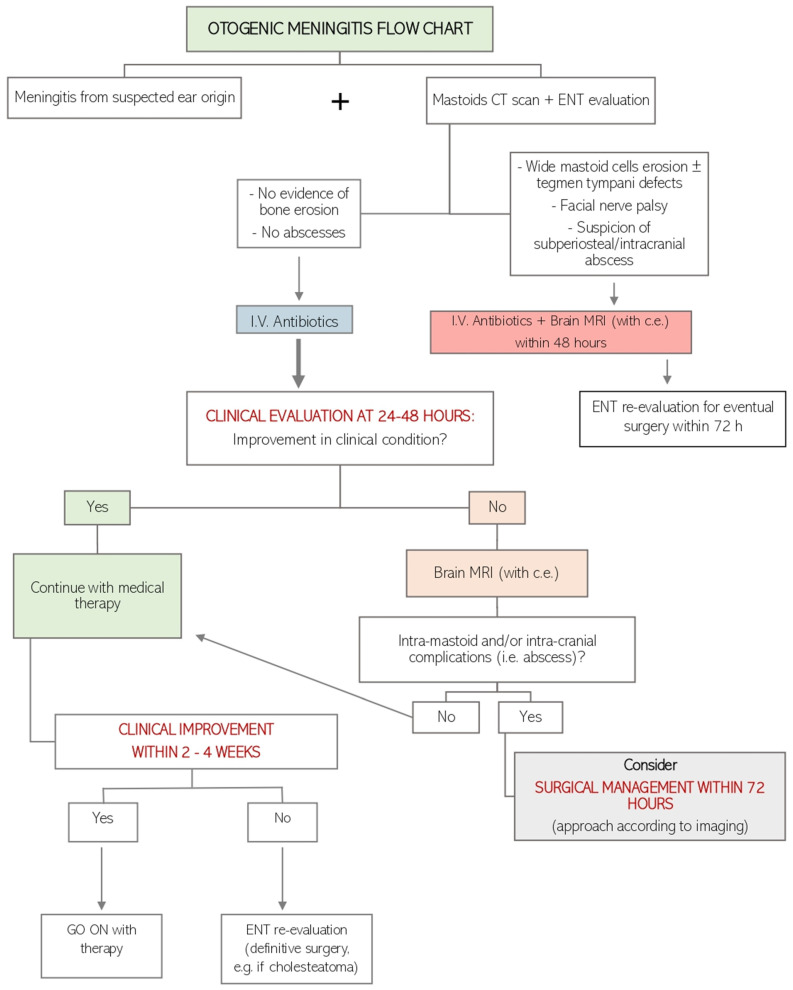
Flow chart of otogenic meningitis management in our clinical practice. I.V.: intravenous; CT: computed tomography; MRI: magnetic resonance imaging.

In our departments, surgical treatment is decided upon based on a patient’s response to medical treatment during the initial 24–48 h and/or magnetic resonance (MRI) findings (Figure 1).

In cases of meningitis of an otogenic source being suspected, mastoid computed tomography (CT) scans are performed to look for any sign of bone erosion and/or purulent collection. In acute otitis media and the re-exacerbation of chronic otitis media, the emergency decision-making algorithm is the same, with the difference lying in the fact that definitive surgical treatment of the latter (i.e., cholesteatoma) might be postponed after the exacerbation is resolved (see later). When intramastoid/cranial abscesses are detected on the first radiological examination, a brain MRI with contrast enhancement is then required for a more accurate evaluation. Intravenous broad-spectrum antibiotic therapy is promptly started in any event, and the patient is strictly monitored from a neurological perspective for the next 24–48 h. Cerebrospinal fluid (CSF) pathogen isolation and culture with antibiotic therapy adjustments according to the newly available antibiogram are advisable. Should a patient’s clinical condition fail to improve within the next 48 h, a brain MRI is mandatory. In the event of complications (i.e., intramastoid and/or intracranial and different from meningitis), a surgical approach based on the radiological findings is necessary within 72 h.

From a surgical perspective, we usually suggest transtympanic drainage (myringotomy or tympanostomy tube insertion) only if a patient’s clinical condition does not improve despite 24–48 h of proper medical therapy. Moreover, in the event of complications (i.e., intramastoid and or intracranial and different from meningitis) being identified on brain MRI or a deterioration in clinical condition despite previous transtympanic drainage, a more invasive surgical procedure, such as mastoidectomy with osteomyelitis bone removal, is indicated.

When the radiological assessments depict pathologies which have to be properly treated by surgery (i.e., cholesteatoma), surgery is planned within 4 weeks or sooner if the clinical neurological findings worsen.

### 2.3. A Literature Review of Otogenic Meningitis from Acute Otitis Media

**Figure 2:**
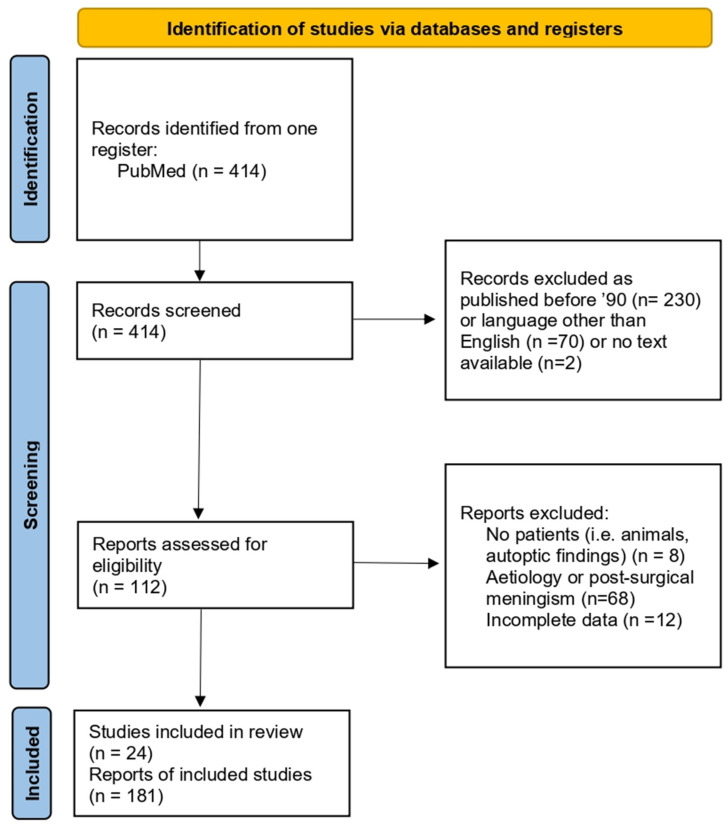
PRISMA flow chart (Page MJ, McKenzie JE, Bossuyt PM, Boutron I, Hoffmann TC, Mulrow CD, et al. *The PRISMA 2020 statement: an updated guideline for reporting systematic reviews*. BMJ 2021;372:n71. doi: 10.1136/bmj.n71).

A comprehensive review of the literature was then conducted to identify and compare articles pertaining to otogenic meningitis from acute otitis media. The Preferred Reporting Items for Systematic Reviews and Meta-Analysis (PRISMA) checklist and statement recommendations guided the qualitative systematic review process (Figure 2). Hence, we conducted an electronic search on PubMed to gather relevant publications regarding this topic. The search strategy incorporated key terms related to acute complications of otitis media, meningitis with otogenic findings, and pertinent Medical Subject Headings (MeSH terms), including “Otogenic meningitis” associated with “acute otitis media” or “mastoid opacification”. Additionally, we identified further publications by scrutinizing the reference lists of the articles found in the aforementioned search.

The main purpose of the present study was to analyze data from the literature specifically addressing otogenic meningitis to delineate the clinical features, treatment approaches, and outcomes, aiming to delineate comprehensive treatment guidelines regarding surgical timing and approach. The study population encompassed both adult and pediatric individuals diagnosed with otogenic meningitis. All forms of study designs available in English with a title and abstract and their full manuscript accessible were included.

Other etiologies, such as neoplasms or inner ear malformations, post-surgical and specifically post-cochlear implant meningitis, chronic meningitis, other otogenic intracranial complications as single entities (e.g., subdural empyema, brain abscesses, cerebellar abscesses, lateral/sigmoid sinus thrombosis), and post-traumatic meningitis, were excluded.

Following the initial selection of the titles and abstracts, the full publications were retrieved, along with the available data. The articles were evaluated for various variables, including study size, the main outcome measures, and conclusions. The data were then extracted and organized using an electronic data extraction form. As much as possible, the individual variables related to each patient, concerning the treatment modalities, pathogens isolated from their cerebrospinal fluid, radiological and/or intraoperative findings, outcomes, and follow-up, were standardized to allow for the most accurate comparisons.

## 3. Results

### 3.1. Our Surgical Experience in Otogenic Meningitis

**Figure 3:**
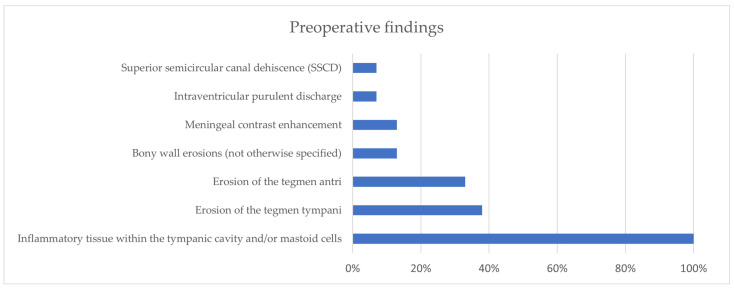
Preoperative radiological findings (CT and/or MRI).

**Figure 4:**
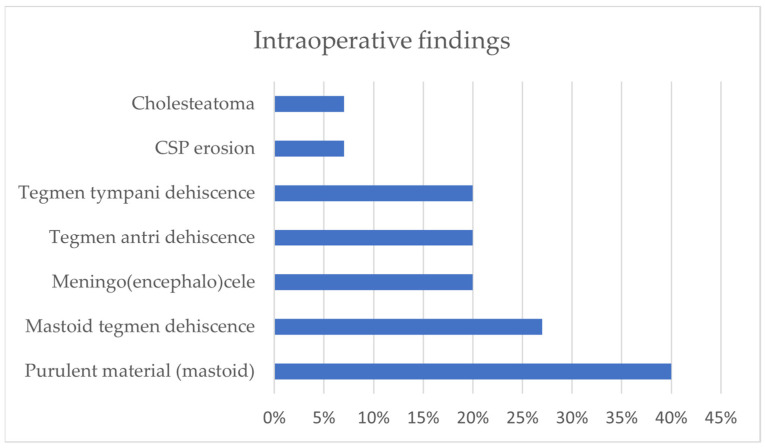
Intraoperative findings.

From the analysis of our comprehensive case series (*n* = 15), the geographical distribution of the conditions included 11 cases from Modena University Hospital and 4 cases from Verona University Hospital. The average age of the enrolled subjects was 59 years (30–77). Comorbidities, which were available for the former 11 patients, included hypertension (*n* = 7, 64%), dyslipidemia (*n* = 5, 45%), and diabetes (*n* = 5, 45%). Among the non-ENT symptoms presented by the patients, the most common were altered consciousness (*n* = 9, 60%), fever (*n* = 8, 53%), headache (*n* = 6, 40%), nuchal rigidity (*n* = 5, 33%), vomiting (*n* = 4, 27%), and aphasia/dysarthria (*n* = 3, 20%). On the other hand, their ENT symptoms included mostly earache (*n* = 10, 67%) and otorrhea (*n* = 3, 20%), while bulging or hyperemia of the tympanic membrane (respectively, 5/15, 33% and 4/15, 27%) was the most commonly found sign; the tympanic membrane was not assessable (due to edema of the external auditory canal) in some patients (*n* = 3, 20%). Among the pathogens isolated, S. Pneumoniae accounted for the majority of cases (9/15, 60%); some isolates came from liquor sampling (8/15, 53%), and S. hominis was then detected in 1 out of 15 patients (7%), while in 6/15 cases, no pathogen was detected (40%). The preoperative radiological findings included inflammatory tissue in the tympanic cavities and mastoid cells (*n* = 15, 100%), erosion of the tegmen tympani (*n* = 6, 38%), and erosion of the tegmen antri (*n* = 5, 33%) (Figure 3). The surgical procedures performed included cortical mastoidectomy (*n* = 15, 100%), tegmen repair (*n* = 9, 60%), and other interventions. Additionally, it was noted that 6/15 patients (40%) showed signs of erosion of the tegmen tympani or antri on preoperative CT scans, even if among the entire study population, 9 individuals (60%) underwent tegmen repair surgery. In further detail, in 6/9, the defect was repaired through a transmastoid approach (67%), and in 2/9, repairs were made through a mini-craniectomy approach (22%), while in 1/9 (11%), a middle cranial fossa approach was applied. The intraoperative findings highlighted the presence of purulent material in most cases (*n* = 6, 40%), as well as tegmen dehiscence, meningoencephalocele (*n* = 3, 20%), cholesteatoma (*n* = 1, 7%), and others (Figure 4). Lastly, it was observed that most of the patients underwent surgery within 7 days of admission (*n* = 11, 73%), and in more than half of cases, this interventional approach occurred within the first 3 days (*n* = 7). In 1 out of 15 cases, bilateral mastoidectomy was required (7%).

### 3.2. Literature Review of Otogenic Meningitis from Acute Otitis Media

Four-hundred and fourteen articles were identified from the database searching process. Out of these, 300 articles were excluded during the screening phase for the following reasons: 230 articles were published before 1990, 70 articles were written in languages other than English, 2 articles did not have any available text, 8 were conducted on animals or models, 68 were not relevant since they regarded different etiologies or concerned secondary meningitis, and 12 had extremely incomplete data. As result, 24 studies, including case reports (*n* = 16) and case series (*n* = 8) with or without reviews published between 1990 and 2023, were included. The overall analysis involved a total of 181 patients (Figure 2).

Excluding studies with titles referencing specific age groups or pathogens typically associated with children and adolescents, such as Fusobacterium Necrophorum, the overall mean age was found to be 38.5 years. Regarding the symptomatology, considering only patients whose symptoms were reported (*n* = 72), the prevalence rates were as follows: otalgia (56%), otorrhea (21%), headache (69%), fever (53%), nausea/vomiting (43%), nuchal rigidity (58%), and altered consciousness (38%). Similarly, two clinical signs were most frequently detected, when present: an erythematous and/or bulging tympanic membrane (60%) and otomastoiditis (12.5%). The most frequently isolated pathogen was S. Pneumoniae, followed by all other pathogens, collectively accounting for approximately 20% of cases. In a considerable proportion of cases, no specific pathogen could be isolated. Due to the limited occurrence of other pathogens in the database, their proportional representation was deemed negligible. Regarding the preoperative computed tomography (CT) findings, nearly all the patients exhibited opacification of the middle ear and mastoid cavity, with temporal osteomyelitis present in 41% of cases (which appeared more prevalent in children), tegmen defects in 26% of cases, and indefinite evidence of intracranial air bubbles in 22% of cases. The average interval between diagnosis and intervention was found to be 7 days. Surgical interventions, which were performed in 45 subjects overall, commonly included myringotomy, in 83% of cases; tympanostomy tube insertion, in 34% of cases; and various degrees of mastoidectomy, in 33% of cases. Interestingly, five individuals (11.1%) required more than one surgery, including one patient in whom the initial surgical procedure involving myringotomy was followed by a second stage with tympanostomy tube insertion (TTI), and a third surgical stage at 5 months with a subtotal petrosectomy plus craniotomy due to persistent cerebrospinal fluid leakage. In another patient, the primary surgical intervention with myringotomy was followed by secondary TTI, while in three other patients, myringotomy with TTI during the first procedure was succeeded by canal wall up (CWU) mastoidectomy. In 13 of the selected articles, neurologic and/or otologic sequelae were reported, with a satisfactory outcome free from long-term complications described in 7/13 studies (patient *n* = 12). Otologic sequalae included conductive hearing loss in 2 case reports (patient *n* = 2), which were resolved through delayed ossiculoplasty, and profound sensorineural hearing loss in 2 individuals, one of whom experienced this following Fusobacterium-caused otogenic meningitis [12]. From a neurologic perspective, hemiplegia was the most commonly detected long-term complication (*n* = 7), followed by cranial nerve palsies (*n* = 2) and hydrocephalus (*n* = 2).

## 4. Discussion

In the literature, it has been noted that approximately 58% of all-cause adult meningitis can be traced back to an otogenic source. An ear infection has the potential to spread, reaching the subarachnoid space either through bloodstream dissemination or direct invasion. Typically, the infection progresses through demineralized bone during acute infection or via resorption caused by cholesteatoma or osteitis in chronic destructive disease. Additionally, retrograde thrombophlebitis of the lateral sinus may occur, facilitated by the spread of infected thrombi through pre-existing anatomical pathways. These pathways include the oval or round window, the internal auditory canal, the cochlear and vestibular aqueducts, congenital bony defects like those in the facial canal, or acquired defects resulting from fractures, neoplasms, stapedectomy, or implantable devices [10,14].

A tegmen tympani defect might provide a route for otogenic intracranial sepsis [15,16]. In the presence of symptoms and signs typical of meningitis, the pathogen is isolated by cerebrospinal fluid (CSF) culture. Otogenic meningitis is sometimes associated with other processes and complications, such as brain abscesses, which require a different management strategy, usually devised in combination with a neurosurgeon specialist [17]. In some cases, the helpful procedure of CSF sampling via lumbar puncture is contraindicated when other complications which could lead to transtentorial herniation coexist (e.g., if large posterior abscesses are associated). While pathogens are typically isolated from cerebrospinal fluid (CSF) for diagnostic purposes, additional cultures may be conducted if otorrhea is present or if the patient undergoes procedures such as myringotomy or mastoidectomy. However, these subsequent cultures are often unreliable due to the patient usually having already commenced treatment with a broad-spectrum antibiotic that covers these pathogens. In Marom and colleagues’ study, 50% of patients had identical CSF and ear culture results, but the remaining 50% had contaminant or no growth in their ear cultures [9].

Although a tegmen defect may sometimes be identified in asymptomatic patients on CT scans, as well as post mortem on autopsy, tegmen tympani defects are found in 15–35% of patients who present with otorrhea [18]. Approximately 80% of skull bone defects within the region of the middle and posterior cranial fossa remain asymptomatic and usually emerge incidentally [1].

Within the assessment of otogenic meningitis, a radiological assessment with CT and magnetic resonance imaging (MRI) scans is crucial, as they both provide relevant etiological information at the level of the middle ear and mastoid. Indeed, the guidance relies on their importance in deciding on an adequate treatment, which can be a combination of medical and surgical or solely medical approaches. High-resolution CT scans of the temporal bone show the tympanic cavity’s and/or mastoid’s status in terms of bony defects, bone tissue reactions, and intracranial complications (herniation, hydrocephalus, empyema, otogenic pneumocephalus, venous or arterial infarction, and abscess formation). MRI is superior to CT in visualizing otogenic parenchymal abscesses, dural enhancement, nearby subdural empyema, cerebritis, labyrinthitis, retrocochlear or intracranial abnormalities, and meningoencephalocele. Moreover, contrast-enhanced T1-weighted images have higher specificity and the potential to detect parenchymal-associated abnormalities [1,19,20]. Compared to CT scans, MRI can also reduce the radiation exposure risk in children [20]. Magnetic resonance imaging can also show spontaneous CSF fistulas between the subarachnoid space and middle ear or defects in the osseous labyrinth. Hence, due to such detailed potential, MRI represents the main imaging technique for assessing middle ear infective complications leading to CSF fistulas, meningitis, or cerebral abscess formation [21,22]. However, the use of a CT scan is still preferred over the use of magnetic MRI for some issues, such as bony defects or for the diagnosis of pneumocephalus [23]. As reported by Bruschini et al., the sensitivity and specificity of any imaging technique in identifying tegmen tympani dehiscence appeared to be 63.2% and 71.4%, respectively, while for mastoid erosion, these values were 66.7% and 100%, respectively, and lower rates were evidenced for meningoencephalocele (14.3% and 100%, respectively) [1].

### Otogenic Meningitis

Although nowadays the risk of a complicated middle ear infection is relatively low, this danger still exists, both in adult and pediatric populations [24]. Barry et al. described equal dominance of acute and chronic otitis media as etiological factors in intracranial infection in an 18-year French series [25]. However, most twentieth-century adult series have demonstrated the predominance of chronic otitis media as a causative factor [26,27,28,29,30]. Conversely, intracranial complications in children appear to be caused more commonly by acute otitis media than the chronic equivalent [31,32,33,34,35,36]. It is widely accepted that otitis media is the most common cause of pediatric meningitis due to the proximity of the tympanomastoid cavity to the dura, as well as the presence of inner ear malformations and the relative ease of the hematogenous spread of the infectious process from the middle ear cavity. Furthermore, the connection between the hematopoietic bone marrow in the temporal bone and the tympanic cavity in autopsies of infants with meningitis and concurrent otitis media has also been suggested as a potential route for infection [9].

Once otogenic meningitis is suspected or diagnosed, starting intravenous broad-spectrum antibiotic therapy as soon as possible is unanimously indicated in the literature, which may be adjusted later according to bacterial cultures, if available [19,37]. For instance, the mortality rate of pneumococcal meningitis varies from 16% to 37%, while neurological sequelae occur in 30–52% of cases [38,39]. Delays in the prompt diagnosis of this potentially severe condition and the proper initiation of antibiotics have been linked to increased mortality [39]. In a retrospective study of otitis-related pneumococcal meningitis, Østergaard and colleagues found that early administration of antibiotic treatment was associated with a lower incidence of sequelae and death [40]. Advanced age, chronic underlying disease, patients with lung foci from pneumococcal infection, and those who presented with seizures were associated with a lower survival rate [41]. Similarly, sequelae were found in 41% of patients, occurring more frequently in those who required assisted ventilation and were not treated with steroids [41].

In spite of these assumptions, the approach to surgical treatment of the ear in patients with otogenic meningitis is discordant. The majority of authors consider conservative treatment with broad-spectrum antimicrobials with adequate central nervous system penetration, along with dexamethasone, the gold standard of care, even if close monitoring for neurological deterioration is widely recommended. Hence, in the literature, surgical intervention at the primary focus (i.e., the ear and/or mastoid cavity) is not considered mandatory, as medical therapy often leads to a full recovery without any neurological sequelae [9,38].

Nevertheless, acute otitis media complicated by meningitis sometimes worsens despite medical therapy. In such cases, a relatively rapid and safe procedure such as myringotomy with or without transtympanic drainage placement may be sufficient to drain the tympanic contents, helping clinical improvement. In Marom et al.’s study, the overall outcomes of adult otogenic meningitis patients being treated with intravenous antibiotic therapy were successful, and only a few cases required surgical interventions, which were minor in most cases (i.e., sole myringotomy) [9].

As an example, Damergis et al. described a case of a 33-year-old man with pneumococcal meningitis and venous sinus thrombosis and confirmed the presence of bilateral mastoid fluid, as well as bilateral maxillary sinus disease. Due to the fact that his fever and neurological symptoms did not improve upon him receiving intravenous antibiotics, the patient underwent bilateral myringotomies, which were followed by a significant improvement [41]. Bilaterality of a more invasive surgery is feasible, as this was performed in 1 out of 15 patients in our series and as reported by Job and colleagues too, who describe bilateral simultaneous hearing conservation mastoidectomy in three patients [42]. 

Gaudin reported a case of invasive pneumococcal disease and concurrent COVID-19 infection with otogenic meningitis in a patient with a right tegmen tympani defect and serous effusion, consistent with acute otitis media. The authors performed an emergent myringotomy with grommet placement, following which the patient was successfully discharged, with home-setting antibiotic therapy for a total of six weeks [43].

Lastly, as intuited from a case report of polymicrobial bacterial meningitis with concurrent chronic suppurative otitis media described by Vata and colleagues, worsening of the clinical evolution and biological findings, when unresolved even through broad-spectrum antimicrobial therapy, dictates a surgical approach [10].

As mentioned above, approximately 80% of skull base bony defects within the region of the middle and posterior cranial fossa remain asymptomatic and thus are usually demonstrated incidentally [1]. However, their role in symptomatic and complicated otitis media increases and pushes for more invasive surgical intervention. In acute otitis media complicated by meningitis, indication of mastoidectomy or another surgical intervention is very rare and is reserved for cases showing signs of osteomyelitis or bony defects or those that do not clinically improve despite myringotomy or grommet placement.

When it is not performed on admission, surgical intervention is mostly indicated due to a deterioration in clinical status or when any wide mastoid cell erosion with or without dehiscence (i.e., tegmen antri, tegmen tympani, sigmoid sinus, etc.) is detected on temporal bone CT scans. Hence, surgical evacuation with purulent discharge and granulation removal, as well as the concurrent repair of bony defects, appears to be mandatory in order to restore compartment sealing, as well stated by Slovik and colleagues [13]. The rationale behind urgent intervention in cases of clinical status deterioration, either myringotomy or mastoidectomy, is that the middle ear and mastoid cavity constitute bacterial sources that promote meningeal invasion and that by eradicating such sources, further seeding into the CSF is disrupted. Hence, prompt intervention promotes a rapid recovery, especially in cases of unresponsiveness to medical therapy [44,45]. In this scenario, CT scans offer reliable detection of otitis complications with a reported sensitivity of up to 97% [46]. Higher suspicion should be upheld in high-risk patients, including newborn, diabetic, elderly, immunosuppressed, or debilitated patients, as in such cohorts of subjects, prompt surgical intervention might be indicated to anticipate further complications [47,48,49].

The data derived from our case series reveal that our current management of otogenic meningitis appears to be in accordance with that in the literature. Once this diagnosis has been suspected, empiric treatment is promptly administered via intravenous broad-spectrum antibiotics, along with systemic corticosteroids, while waiting for culture of and an antibiogram test on CSF. Next, an ENT evaluation with ear and brain CT and MRI scans is carried out to understand the underlying etiological focus better (acute/chronic otitis media or other).

As acute otitis media usually shows an unspecific tympanic–mastoid effusion on CT and/or MRI, the medical treatment resolves both ear discharge and the cause of the meningitis, too. Hence, such patients are maintained under strict neurological monitoring to look at their clinical evolution and systemic therapy within the first 48–72 h. If their clinical condition worsens, transtympanic drainage can be performed easily and promptly (usually within 7 days from the diagnosis in order to drain any tympanic–mastoid collection). The finding of isolated mastoid cell erosion alone does not indicate a surgical approach, as no surgery is indicated in cases of clinical recovery with antibiotic and corticosteroid therapy. However, if bone erosion (e.g., erosion of the mastoid cells) is noticed on CT and a patient’s clinical conditions do not improve, a surgical procedure with mastoidectomy and the removal of the osteomyelitis bone is required [50]. Furthermore, if other pathologies which can be treated by surgery are detected on CT/MRI (e.g., cholesteatoma, meningocele, or meningoencephalocele), surgery ideally has to be planned within 4 weeks, or sooner in cases of clinical worsening or recrudescence.

## 5. Conclusions

Timely evaluation by an otolaryngologist is crucial for all meningitis cases to rule out an ear-related cause. Antimicrobial therapy is the primary treatment for acute bacterial meningitis, with surgery being reserved for the event of complications, as well as for those who do not respond adequately to such initial treatment within 48 h, in order to sterilize the tympanic and mastoid cavity and thus the suspected infective foci. The management options range from myringotomy with or without grommet insertion to mastoidectomy. Prompt CT and MRI scans are indicated based on the clinical course, and a subsequent radiological assessment is recommended if recurrent infection-causing defects are suspected.

## Data Availability

The original contributions presented in this study are included in the article; further inquiries can be directed to the corresponding author.
